# Hospital Payments Increase as Payments to Surgeons Decrease for Common Inpatient Orthopaedic Procedures

**DOI:** 10.5435/JAAOSGlobal-D-20-00026

**Published:** 2020-04-01

**Authors:** Majd Marrache, Andrew B. Harris, Varun Puvanesarajah, Micheal Raad, Hamid Hassanzadeh, Uma Srikumaran, James R. Ficke, Joseph F. Levy, Amit Jain

**Affiliations:** From the Department of Orthopaedic Surgery, The Johns Hopkins University School of Medicine, Baltimore, MD (Dr. Marrache, Mr. Harris, Dr. Puvanesarajah, Dr. Raad, Dr. Srikumaran, Dr. Ficke, Dr. Jain); the Department of Orthopaedic Surgery, University of Virginia School of Medicine, Charlottesville, VA (Dr. Hassanzadeh); and the Department of Health Policy and Management, Johns Hopkins Bloomberg School of Public Health, Baltimore, MD (Dr. Levy).

## Abstract

As healthcare costs continue to increase in the United States, it is important to understand the trends in the allocation of healthcare spending for common orthopaedic surgical procedures. We investigated the recent trends in (1) total net payments (for episode of care), (2) payments to hospitals, (3) payments to physicians, (4) payments to physicians as a percentage of total net payments, and (5) regional variation in hospital and physician payments for four common orthopaedic procedures.

**Methods:**

Using a private insurance claims database, we analyzed the payments to US hospitals and physicians from 2010 to 2016 for primary total hip arthroplasty (THA) (n = 128,269), total knee arthroplasty (TKA) (n = 223,319), 1-level anterior cervical diskectomy and fusion (ACDF) (n = 51,477), and 1-level lumbar-instrumented posterior spinal fusion (PSF) (n = 45,680). Regional variations in payments were also assessed. Trends were analyzed using linear regression models adjusting for age, sex, comorbidities, duration of hospital stay, and inflation (alpha = 0.05).

**Results:**

Inflation-adjusted total net payments for the episode of care increased by the following percentages per year: 5.2% for ACDF, 3.2% for PSF, 2.9% for TKA, and 2.6% for THA. Annual inflation-adjusted hospital payments increased significantly for all 4 procedures, whereas annual inflation-adjusted physician payments decreased by −2.2%/year for PSF, −1.5%/year for TKA, −1.1%/year for THA, and −0.4%/year for ACDF (all, *P* < 0.001). As a percentage of total net payments, physician payments decreased markedly for ACDF (−4.6%), PSF (−3.1%), TKA (−2.1%), and THA (−1.8%). Hospital and physician payments varied significantly by region and were both highest in the West (*P* < 0.001).

**Conclusions:**

From 2010 to 2016, inflation-adjusted total net payments for 4 common orthopaedic surgical procedures increased markedly, as did payments to the US hospitals for these procedures. Payments to orthopaedic surgeons for these procedures decreased markedly during the same period.

Healthcare costs continue to increase in the United States, and elective orthopaedic surgical procedures account for a substantial portion of these costs.^1-3^ In addition to the increasing costs of hospitalization and postacute care, factors such as novel implants, osteobiologic agents, and the aging US population contribute to increasing expenses in orthopaedic surgical care.^4-6^

Traditional fee-for-service payment models are being replaced by alternative reimbursement strategies that link reimbursement to patient outcomes in an effort to curb increasing costs and improve the quality of health care.^7^ Some recent cost-containment efforts have also reduced physician reimbursement for the services rendered.^8,9^ The effect of these strategies on the hospital-physician relationship remains unclear.^10-12^

The aims of our study were to investigate the recent trends in (1) total net payments (for episode of care), (2) payments to hospitals, (3) payments to physicians, (4) payments to physicians as a percentage of total net payments, and (5) regional variation in hospital and physician payments for four common orthopaedic procedures.

## Methods

This study did not involve the use, collection, or distribution of individually identified data and was exempt from institutional review board approval. The authors did not receive any funding or grants in support of their research for or preparation of this work.

### Data Source

We conducted a retrospective review of the MarketScan Commercial Claims and Encounters Database (Truven Health Analytics) from January 1, 2010, through December 31, 2016. The MarketScan database provides person-specific data on clinical utilization, expenditures, and enrollment for approximately 51 million patients aged younger than 65 years. The database includes health claims stored in a Health Insurance Portability and Accountability Act—compliant format that are from individuals with commercial employer-sponsored health insurance or Medicare.^13^ Individual claims are linked to codes from the *International Classification of Diseases, Ninth and Tenth Revisions,* and the Current Procedural Terminology. The data contain inpatient claims, outpatient claims, and prescription drug claims. We queried the inpatient claims database to identify our cohort.

### Study Population

**Figure 1:**
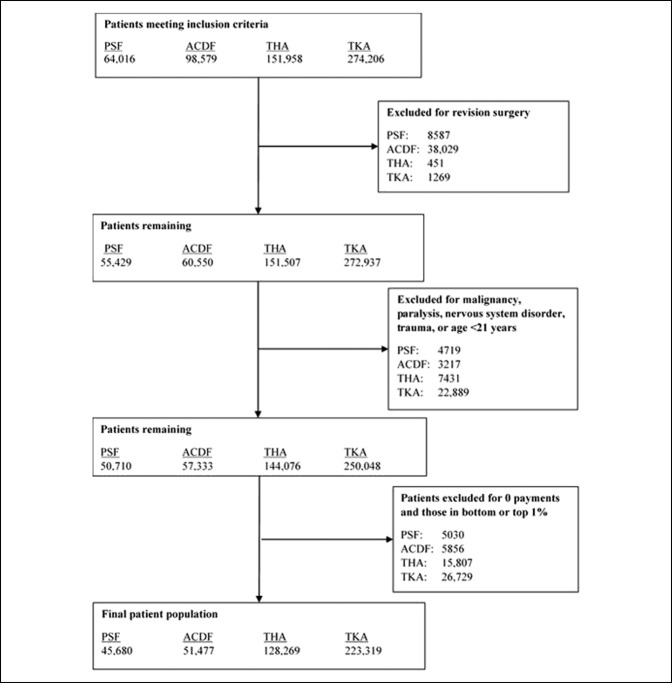
Flowchart showing the exclusion of patients from the initial inclusion cohort and the final sample used in the study. ACDF, 1-level anterior cervical diskectomy and fusion; PSF, 1-level lumbar instrumented posterior spinal fusion; THA, total hip arthroplasty; TKA, total knee arthroplasty.

We included 448,745 patients who underwent the following primary elective procedures: total knee arthroplasty (TKA) (n = 223,319), total hip arthroplasty (THA) (n = 128,269), 1-level anterior cervical diskectomy and fusion (ACDF) (n = 51,477), or 1-level lumbar-instrumented posterior fusion (PSF) (n = 45,680) from 2010 to 2016 (Table 1). The Current Procedural Terminology codes and the *International Classification of Diseases, Ninth and Tenth Revisions,* codes were used to select our study cohorts. To avoid potential confounding variables, we excluded patients with a diagnosis of primary or secondary malignancy, neoplasms, trauma, or disorders of the nervous system who underwent these procedures (Appendix Tables I to III, http://links.lww.com/JG9/A71, http://links.lww.com/JG9/A72, http://links.lww.com/JG9/A73, respectively, Figure 1).

**Table 1:** Characteristics of 448,745 Patients Who Underwent Four Common Orthopaedic Procedures, MarketScan Commercial Claims and Encounters Database, 2010 to 2016

Characteristic	N (%)			
	ACDF (N = 51,477)	PSF (N = 45,680)	THA (N = 128,269)	TKA (N = 223,319)
Age, (yr)	50 (44-56)^a^	53 (46-59)^a^	57 (52-61)^a^	59 (54-62)^a^
Female sex	28,086 (55)	26,265 (57)	61,515 (48)	133,149 (60)
Employment status				
Full-time	19,439 (38)	16,506 (36)	44,563 (35)	73,083 (33)
Part-time	319 (0.62)	239 (0.52)	977 (0.76)	1378 (0.62)
Early retiree	2392 (4.6)	3212 (7.0)	12,148 (9.5)	24,295 (11)
Other^b^	29,327 (57)	25,723 (56)	70,581 (55)	124,563 (56)
Region				
Northeast	8087 (16)	6809 (15)	29,007 (23)	40,915 (18)
Northwest	10,330 (20)	11,535 (25)	33,306 (26)	61,883 (28)
South	24,095 (47)	20,328 (45)	43,066 (34)	84,870 (38)
West	8360 (16)	6134 (13)	20,181 (16)	30,872 (14)
Unknown	605 (1.2)	874 (1.9)	2709 (2.1)	4779 (2.1)
Duration of hospital stay (d)	1 (1-1)^a^	3 (2-3)^a^	2 (2-3)^a^	3 (2-3)^a^
Hospital payment (1000$)^c^	21 (14-20)^a^	41 (30-56)^a^	24 (18-31)^a^	23 (17-31)^a^
Physician payment (1000$)^c^	4.9 (3.7-6.3)^a^	5.7 (4.5-7.5)^a^	2.2 (1.7-2.8)^a^	2.3 (1.8-2.9)^a^

ACDF = 1-level anterior cervical diskectomy and fusion, PSF = 1-level instrumented lumbar posterior spinal fusion, THA = total hip arthroplasty, TKA = total knee arthroplasty

a Presented as median (interquartile range).

b Values rounded to two significant digits.

c Includes retiree, Medicare-eligible retiree, COBRA (Consolidated Omnibus Budget Reconciliation Act) continue, long-term disability, and surviving spouse/dependent.

### Outcomes of Interest

The primary outcomes of interest were trends in total net payments, hospital payments, physician payments, and physician payments as a percentage of total net payments. We defined total net payments as payments to the surgeon plus payments to the hospital. Hospital payments were defined as net payments made to a hospital for services provided during a single admission. Physician payments were defined as net payments made solely for services provided by the primary surgeon. Our secondary outcome was variation in payments across the US geographic regions. Regions, as defined by the US Census Bureau,^14^ were assigned according to the state in which the surgery was performed. All payments excluded deductibles, copayments, coinsurance, and coordination of benefits. Payment outliers and coding errors, defined as payments in the 1st and 99th percentiles for each procedure, were excluded from the analysis. All payments were adjusted for inflation to 2016 US dollars using the US Bureau of Labor Statistics Consumer Price Index.^15^

### Statistical Analysis

Descriptive statistics for continuous variables are reported as mean (±SD) or median (interquartile range), depending on the normality of data, and categorical variables are reported as number (percentage), unless otherwise specified. We used analysis of variance with a Tukey multiple comparison test to determine the regional differences in hospital and physician payments. The Tukey test can be used to determine which means in a set of means differ from the others. A multivariable linear regression analysis was performed to assess incremental changes in total net payments, hospital payments, physician payments, and physician payments as percentages of total net payments over time for each procedure. We calculated the Elixhauser Comorbidity Index value for each patient as described by Quan et al.^16^ All multivariable regression outcomes were adjusted for age, sex, duration of hospital stay, Elixhauser value, and region in which the surgery was performed. Significance was set at *P* < 0.05, and robust standard errors were computed. All statistical analyses were conducted using SAS, version 9.4, software (SAS Institute, Cary, NC).

## Results

### Total Net Payment

We found significant annual increases in the total net payments for all 4 procedures: for ACDF, 3.6%/year; for TKA, 2.4%/year; for PSF, 2.2%/year; and for THA, 2.1%/year (Table 2).

**Table 2:** Annual Changes^a^ in Hospital and Physician Payments for Four Orthopaedic Surgical Procedures, MarketScan Commercial Claims and Encounters Database, 2010 to 2016

Surgical Procedure	Hospital Payments		Physician Payments		Total Net Payments	
	Mean (95% CI) Payment, $	Change, %	Mean (95% CI) Payment, $	Change, %	Mean (95% CI) Payment, $	Change, %
ACDF	989 (932-1045)	5.2	−26 (−36 to −16)	−0.4	952 (891-1013)	3.6
PSF	1310 (1209-1411)	3.2	−154 (−165 to −143)	−2.2	1137 (1032-1242)	2.2
TKA	708 (682-733)	2.9	−40 (−42 to −38)	−1.5	666 (640-692)	2.4
THA	647 (615-679)	2.6	−28 (−31 to −25)	−1.1	611 (578-644)	2.1

ACDF = 1-level anterior cervical diskectomy and fusion, CI = confidence interval, PSF = 1-level lumbar-instrumented posterior spinal fusion, THA = total hip arthroplasty, TKA = total knee arthroplasty

a *P* < 0.001 for all mean changes in payments. Adjusted for patient age, sex, comorbidities, duration of hospital stay, and the US region.

### Hospital Payments

Annual inflation-adjusted hospital payments increased significantly for all 4 procedures: by 5.2% for ACDF, 3.2% for PSF, 2.9% for TKA, and 2.6% for THA (all, *P* < 0.001; Table 2). When comparing 2010 to 2016 after adjusting for confounders, hospital payments also increased significantly for all 4 procedures (all values rounded to two significant digits): a 7-year increase for PSF, $7900 (95% confidence interval [CI]: $7100 to $8700); for ACDF, $6200 (95% CI: $6000 to $6700); for TKA, $4200 (95% CI: $3900 to $4400); and for THA, $3800 (95% CI: $3600 to $4100) (all, *P* < 0.001).

### Physician Payments

**Figure 2:**
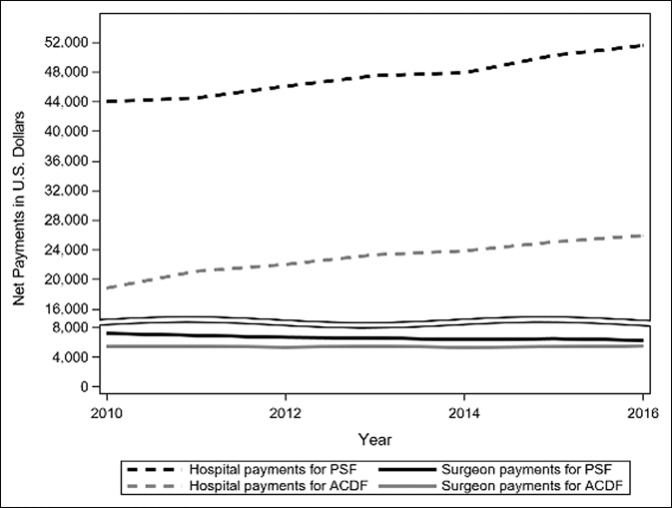
Graph showing the trends in hospital and physician payments in 1-level lumbar-instrumented posterior spinal fusion (PSF) and 1-level anterior cervical diskectomy and fusion (ACDF), 2010 to 2016, from the MarketScan Commercial Claims and Encounters Database.

**Figure 3:**
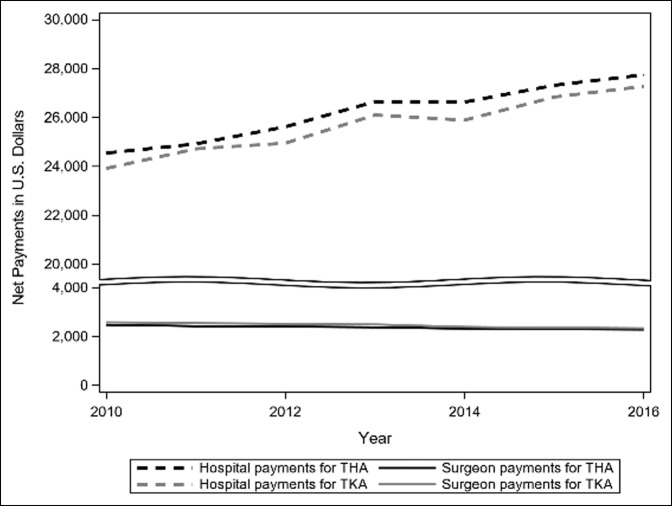
Graph showing the trends in hospital and physician payments in total hip arthroplasty (THA) and total knee arthroplasty (TKA), 2010 to 2016, from the MarketScan Commercial Claims and Encounters Database.

During the same period, significant decreases were observed in the annual inflation-adjusted physician payments for all 4 procedures: for PSF, −$154/year (95% CI: −$165 to −$143); for ACDF, –$26/year (95% CI: −$36 to −$16); for TKA, −$40/year (95% CI: −$42 to −$38); and for THA, −$28/year (95% CI: −$31 to −$25) (Table 2 and Figures 2 and 3).

### Physician Payments as a Percentage of Total Net Payments

**Figure 4:**
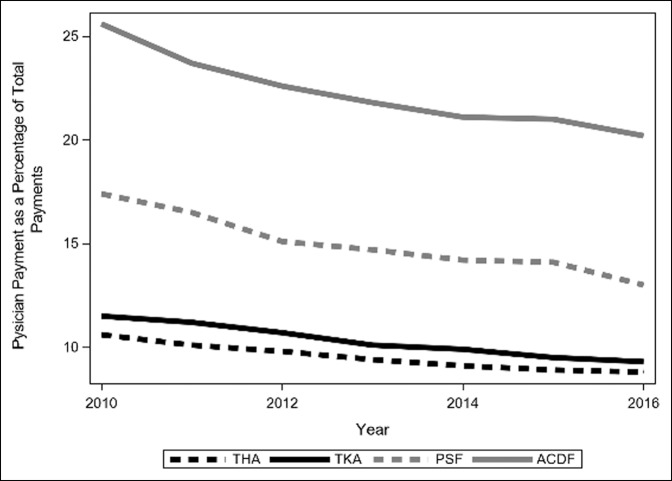
Graph showing the trends in physician payments as a percentage of total net payments for total hip arthroplasty (THA), total knee arthroplasty (TKA), 1-level lumbar-instrumented posterior spinal fusion (PSF), and 1-level anterior cervical diskectomy and fusion (ACDF), 2010 to 2016.

From 2010 to 2016, physician payments as a percentage of total net payments decreased significantly for all 4 procedures: by −4.6% for ACDF (95% CI: −5.0% to −4.2%), by −3.1% for PSF (95% CI: −3.3% to −2.8%), by −2.1% for TKA (95% CI:–2.2% to −2.0%), and by −1.8% for THA (95% CI: −1.8% to −1.6%) (Figure 4).

### Regional Variation

Hospital and physician payments varied markedly by the US region. Hospital payments were highest in the West and lowest in the Northwest (mean difference, $3900; 95% CI: $3700 to $4100; *P* < 0.001). Physician payments were highest in the West and lowest in the South (after the “unknown” region) (*P* < 0.001; Table 3).

**Table 3:** Regional Differences in Hospital and Physician Payments for Four Types of Orthopaedic Surgical Procedure Combined, MarketScan Commercial Claims and Encounters Database, 2010 to 2016

Region	No. of Cases	Hospital Payments		Physician Payments	
		Mean ± SD, $	*P*	Mean ± SD, $	*P*
Northeast	84,818	26,349 ± 14,147	<0.001^a^	3,223 ± 2,505	0.001^b^
Northwest	117,054	25,955 ± 13,553		3,299 ± 2,568	
South	172,359	26,941 ± 15,322		3,087 ± 2,150	
West	65,547	29,838 ± 15,772		3,305 ± 2,630	
Unknown	8,967	27,174 ± 13,814		2,795 ± 1,920	

a Significant difference among all regions except for South and Unknown.

b Significant difference among all regions except for Northwest and West.

## Discussion

From 2010 to 2016, total net payments for the episode of care for four common orthopaedic procedures (ACDF, PSF, THA, and TKA) increased markedly. During the same period, physician payments for these procedures decreased annually, whereas hospital payments increased.

These results have several implications. First, they show that the decrease in physician payments did not result in an overall decrease in episode-of-care costs. Second, the aging US population has an increasing comorbidity burden, which is projected to grow over time in both total joint arthroplasty and spine surgery.^17,18^ Thus, orthopaedic surgeons are expected to treat more patients and receive proportionally less of the total net payment for services rendered. The effects of these factors on surgeon fatigue and burnout are unclear. Third, although the goal of the new payment models is to emphasize value over volume in health care, reducing physician payments may lead to an unintended increase in the volume of procedures performed by a surgeon to achieve the same compensation earned under previous payment models. Finally, the effect of decreasing physician payments as a percentage of total net payments on the hospital-physician relationship is unclear. The idea of “physician-hospital alignment” has been studied extensively in the field of orthopaedic surgery, and the hospital-physician payment disparity is a growing issue that will likely outlast the current era of healthcare payment reform.^10-12,19^ In a 2013 review by Page et al,^12^ the authors investigated financial, regulatory, healthcare reform, and cultural issues as they relate to physician-hospital alignment in orthopaedic surgery. They found that financial concerns were the most consistently identified factor affecting physician-hospital alignment. Other factors affecting physician-hospital alignment include accountability, clinical authority, and patient advocacy. Poor physician-hospital alignment may lead to poor patient experience and increased rates of readmission and emergency visits, all of which would further increase healthcare costs. Several studies have demonstrated the efficacy of maximizing the physician-hospital alignment to provide high-quality care and lower costs.^12,20^

Although the increase in orthopaedic implant and supply costs with the introduction of new technologies contributes to increasing hospital costs, we posit that the reasons for increasing hospital costs are multifactorial. One major factor may be an increase in indirect costs that are not specifically for services rendered during an admission. Indirect costs may be increased by several factors. First, the number of nonclinical healthcare workers, such as hospital executives and administrative assistants, continues to increase in response to greater regulation and complexity of health care. A recent study estimated that nonclinical healthcare workers, defined as employees with no direct patient contact, cost the system $865 billion US dollars per year.^21^ Second, the wages for hospital executives are increasing. One study found that the wage gap between chief executive officers and orthopaedic surgeons in US nonprofit hospitals almost doubled from 3:1 to 5:1 between 2005 and 2015.^21^ Third, hospitals are increasing their efforts to provide care and referral through multidisciplinary service lines (e.g., musculoskeletal, oncologic, cardiovascular, women's health, and primary care) regardless of whether it is profitable to do so. Development of such service lines may result in increased indirect costs.

Our results are consistent with those of previous studies. A study analyzing National Inpatient Sample claims data from 1996 to 2005 found that, despite a 200% increase in the volume of TKA procedures and the rising cost of hospitalization, inflation-adjusted physician payments decreased by 5% annually during the study period.^8^ In the current study, physician payments decreased similarly for privately insured patients, whereas hospital payments increased. Importantly, physician payments as a percentage of total net payments decreased markedly during our 7-year study period, by 1.8% to 4.6% depending on the procedure.

This study has several limitations. The patients in our study were all covered by private health insurance, and the data reported herein are hospital and physician payments made by private insurance companies. Private payers typically pay more for the same procedures compared with Medicare.^22^ Our results represent population-level trends for patients younger than 65 years, and the trends observed in individual healthcare systems for individual payers may not be representative of all healthcare systems and payers.^23^ In addition, the MarketScan database does not contain granular data on implant or supply costs; thus, the impact of these factors on episode-of-care costs could not be assessed. Finally, although the use of administrative claims data is well accepted in healthcare economics and orthopaedic surgery research, the accuracy of data relies on accurate and consistent coding of diagnoses and procedures.^24^ We addressed this potential limitation by rigorously defining the patient cohort for each procedure according to the methods of similar studies.^25-27^

From 2010 to 2016, inflation-adjusted payments to orthopaedic surgeons decreased markedly, whereas payments to hospitals for common orthopaedic procedures increased markedly. The net result was an increase in total net payments for these procedures. Thus, reducing surgeon payments may not be an effective strategy for reducing the overall episode-of-care costs.
